# Abnormal placental perfusion and the risk of stillbirth: a hospital-based retrospective cohort study

**DOI:** 10.1186/s12884-021-03776-8

**Published:** 2021-04-17

**Authors:** Jiang-Nan Wu, Yun-Yun Ren, Chen Zhu, Ting Peng, Bin Zhang, Ming-Qing Li

**Affiliations:** 1 Department of Clinical Epidemiology, Obstetrics and Gynecology Hospital of Fudan University, 566 Fangxie Rd, Shanghai, 200011 China; 2 Department of Ultrasound, Obstetrics and Gynecology Hospital of Fudan University, Shanghai, China; 3 Department of Obstetrics, Obstetrics and Gynecology Hospital of Fudan University, Shanghai, China; 4 Research Institute, Obstetrics and Gynecology Hospital, Fudan University, Shanghai, China

**Keywords:** Stillbirth, Abnormal placental perfusion, Uterine artery Doppler, Preeclampsia, Retrospective cohort study

## Abstract

**Background:**

A lack of information on specific and interventional factors for stillbirth has made designing preventive strategies difficult, and the stillbirth rate has declined more slowly than the neonatal death rate. We compared the prevalence of stillbirth among the offspring of women with or without abnormal placental perfusion (APP).

**Methods:**

We conducted a hospital-based retrospective cohort study involving women with a singleton pregnancy between 2012 and 2016 (*N* = 41,632). Multivariate analysis was performed to compare the prevalence of stillbirth in infants exposed to APP (defined as any abnormality in right or left uterine artery pulsatility index or resistance index [UtA-PI, −RI] [e.g., > 95th percentile] or presence of early diastolic notching) with that in those not exposed to APP.

**Results:**

Stillbirths were more common among women with APP than among those with normal placental perfusion (stillbirth rate, 4.3 ‰ vs 0.9 ‰; odds ratio (OR), 4.2; 95% confidence interval (CI), 2.2 to 8.0). The association strengths were consistent across groups of infants exposed to APP that separately defined by abnormality in right or left UtA-PI or -RI (OR ranged from 3.2 to 5.3; all *P* ≤ 0.008). The associations were slightly stronger for the unexplained stillbirths. Most of the unexplained stillbirth risk was attributed to APP (59.0%), while a foetal sex disparity existed (94.5% for males and 58.0% for females). Women with normal placental perfusion and a male foetus had higher credibility (e.g., higher specificities) in excluding stillbirths than those with APP and a female foetus at any given false negative rate from 1 to 10% (93.4% ~ 94.1% vs. 12.3% ~ 14.0%).

**Conclusions:**

APP is associated with and accounts for most of the unexplained stillbirth risk. Different mechanisms exist between the sexes. The performance of screening for stillbirth may be improved by stratification according to sex and placental perfusion.

**Supplementary Information:**

The online version contains supplementary material available at 10.1186/s12884-021-03776-8.

## Background

Every year, approximately 2.6 million stillbirths occur worldwide, with a prevalence of 18.4 per 1000 births [1]. The stillbirth rate has declined steadily but more slowly than the neonatal death rate (defined as death within the first 28 days after birth) and more slowly than the rate required to meet the target set to end preventable stillbirths [1–3]. The disparity may be partly attributed to inadequate global attention to the issue and may be associated with a lack of effective preventions since there is neither a unified definition of stillbirth nor a standardized high-coverage and efficient reporting system and since the causes of stillbirth are poorly understood [1, 2, 4].

The systematic evaluation of stillbirths suggests the probable or possible cause in most stillbirths. Obstetric conditions and placental abnormalities are the most common causes of stillbirth, although their distribution differs by race/ethnicity [2, 4]. Thus, examination of placental function might be a measure to balance the risk of stillbirth by early induction of labour or caesarean section [1, 5]. However, without clinical evidence of placental insufficiency (e.g., foetal growth restriction or oligohydramnios), it is difficult to determine whether specific placental abnormalities are associated with stillbirth; thus, the early detection of impaired placental function is still a challenge even in high-income countries [6].

Placental perfusion insufficiency (APP) is frequently accompanied by impaired placental function and is associated with known risk factors for stillbirth, such as maternal preeclampsia (PE) and small for gestational age [7, 8]. Previous studies have indicated that a high uterine artery (UtA) pulsatility index (PI, e.g., > 90th centile) is associated with the risk of stillbirth and that UtA-PI is an important variable in screening for stillbirth [9–12]. However, the association between the risk of stillbirth and APP systematically evaluated by UtA-PI, the UtA-resistance index (RI) and the presence of early diastolic notching (EDN) is still unknown. Furthermore, little is known about the extent of the effect of placental perfusion on the risk of stillbirth compared with the genetic/maternal origin effect. These issues may determine the clinical value of APP in screening for stillbirth. We therefore conducted a retrospective cohort study to clarify these issues.

## Methods

### Study design and data sources

Details of the design have been mentioned in our previous studies [13, 14]. Pregnant women who participated in prenatal examinations at the Obstetrics and Gynaecology Hospital of Fudan University between April 2012 and August 2016 were extracted and followed up till delivery. Basic information (e.g., maternal age at delivery, parity, and residence), pregnancy complications (e.g., gestational diabetes mellitus [GDM] and PE) and outcomes (e.g., foetal sex and stillbirths) was collected during the follow up period [14]. Women with multiple pregnancies and who were lost to follow-up were excluded from the analysis.

### Stillbirths

Stillbirths were defined by Apgar scores of 0 at 1 and 5 min and no signs of life by direct observation for infant foetuses of ≥28 weeks gestation [1, 2] and coded according to the International Classification of Disease (ICD) 10th Revision as O36.401 in the hospital discharge reports.

### APP definition

Placental perfusion of the UtA, including calculation of the left and right UtA pulsatility index (PI), UtA resistance index (RI), and the presence of early diastolic notching (EDN), was measured by sonographers using GE ultrasound devices (GE Healthcare, Zipf, Austria) at 20 to 24 weeks of gestation [14, 15]. Women with a gestational age specific percentile of >95th percentile for the UtA-PI and UtA-RI values was classified as abnormal UtA-PI or -RI perfusion (Table S2) [14]. The presence of EDN was identified by the ISUOG practice guidelines [15]. Pregnant women with any abnormality in the right or left UtA-PI, UtA-RI, or presence of EDN markers were defined as the APP group; otherwise, they were classified as the normal placental perfusion group [14].

### Potential confounders

Potential confounders in the study included PE (yes or no), maternal age at delivery (≤ 24, 25–34, and ≥ 35 years), resident location (local or nonlocal), parity (nulliparous or pluriparous), assisted conception (yes or no), GDM (yes or no) and foetal sex (male or female) [14]. Women who complicated hypertension and proteinuria after 20 weeks of gestation were diagnosed as PE [16]. GDM was diagnosed based on an oral glucose tolerance test at 24 to 28 weeks of gestation [17]. Parity and assisted conception pregnancies were self-reported [13, 14].

### Statistical analysis

The prevalence of stillbirths (including all and unexplained cases) were calculated. Odds ratios (ORs) and 95% confidence interval (CI) for stillbirths were estimated for infants who exposed to APP relative to those without APP exposure. Adjusted ORs were further evaluated in multivariable models in which the potential confounders were included. Sensitivity analyses restricted to women with APP measurement data were conducted. A stratification analysis according to foetal sex was performed to examine potential mechanistic differences between the sexes.

We also estimated the association between variants of APP (separately defined by a single indicator of the six perfusion markers) and the risk of stillbirth. The cumulative risk of stillbirth among women with mild (abnormality in any 1 marker), moderate (abnormalities in any 2 markers) and severe (abnormalities in ≥3 markers) APP subgroups [14] were compared by the Kaplan-Meier curve method. Logistic regression analyses using path analysis models [18] were conducted to quantify the effect size of APP on the risk of stillbirth compared with the effect of PE (presumed to be an effect of maternal origin since PE has recently been considered a maternal origin disorder [19, 20]) [14].

A receiver operating characteristic (ROC) curve analysis was run to determine the performance of the number of weeks between the APP test and delivery in predicting stillbirth. The performance and difference in screening for stillbirth was further assessed by stratification according to the placental perfusion condition (e.g., normal perfusion group and APP group), foetal sex (male or female) and a combination of both. The area under the curve (AUC), sensitivity, specificity and optimal criterion value were estimated under special assumptions (1: the cost of a false negative decision was 100 times that of a false positive decision, and 2: the rate of stillbirth in the sample reflects the real prevalence of stillbirth in the population) and at given false negative rates.

The statistical software packages Stata 12.0, IBM SPSS 22.0 and MedCalc 15.0 were used for the data analyses. A two-sided *P* value < 0.05 was considered statistically significant.

## Results

### Characteristics of the population

Among the 52,047 pregnant women who had pregnancy examinations, 43,473 were followed up until delivery, with a dropout rate of 16.5%. Among them, 41,632 (95.8%) women with single pregnancies were included in the analysis. Most women were local (76.9%), nulliparous (84.9%), and aged 25 to 34 years (85.0%). Only 1.8% of the pregnancies were from assisted conception, and the proportions of women with complicated GDM and PE were 8.4 and 5.7%, respectively (Table 1).

**Table 1:** Prevalence of stillbirth and characteristics based on groups stratified by placental perfusion

Characteristic	Placental perfusion			*P* value ^*^	
	Normal (*N* = 27,720)	Insufficiency (*N* = 3484)	Not available (*N* = 10,428)	Across the three groups	Between normal and insufficiency group
Stillbirth, no. (‰, 95% confidence interval)					
All ^a^	25 (0.9, 0.5–1.3)	15 (4.3, 2.1–6.5)	16 (1.5, 0.8–2.3)	< 0.001	< 0.001
Unexplained^b^	21 (0.8, 0.4–1.1)	14 (4.0, 1.9–6.1)	13 (1.3, 0.6–1.9)	< 0.001	< 0.001
Maternal age at delivery year, no.(%)				< 0.001	0.23
< 25	1250 (5)	142 (4)	717 (7)		
25–34	24,012 (87)	3009 (86)	8374 (80)		
> = 35	2458 (9)	333 (10)	1337 (13)		
Local residence, no.(%)	22,006 (79)	2750 (79)	7263 (70)	< 0.001	0.53
Nulliparous, no. (%)	24,033 (87)	3010 (86)	8284 (79)	< 0.001	0.62
Assisted conception, no. (%)	452 (2)	43 (1)	255 (2)	< 0.001	0.078
Gestational diabetes mellitus, no. (%)	2222 (8)	307 (9)	972 (9)	< 0.001	0.11
Preeclampsia, no. (%)	1370 (8)	398 (11)	599 (6)	< 0.001	0.003
Male sex, no. (%)	14,188 (51)	1860 (53)	5407 (52)	0.037	0.014

^*^ *P* values were derived from chi-square tests or Fisher’s exact tests if applicable

^a^ The numbers for the placental perfusion normal, insufficiency and unavailable groups were 27,724, 3485, and 10,431, respectively

^b^ Stillbirths from terminations of pregnancy due to congenital anomalies were excluded (8 cases)

### Association between APP and the risk of stillbirth

A total of 56 stillbirths occurred during the study period, including 48 cases with unexplained causes (47 cases as antepartum death of unspecified cause and 1 case as intrapartum death of unspecified cause) and 8 congenital malformation-related cases (4 with congenital malformations, 3 with chromosomal abnormalities, and 1 with congenital malformations and chromosomal abnormalities) (Table S1). The prevalence rates of all and unexplained stillbirths were 1.3 (95% CI, 1.0 to 1.7) and 1.2 (95% CI, 0.8 to 1.5) per 1000 births, respectively. The prevalence of stillbirth was higher for infants of women with APP than for those of women with normal perfusion (Table 1).

Table 2 shows the association between APP and the risk of stillbirth. Compared with the foetuses of women with normal perfusion, the foetuses born to women with APP were significantly associated with an increased risk of all stillbirths (adjusted OR, 4.2, 95% CI, 2.2 to 8.0), as well as unexplained stillbirths (adjusted OR, 4.6, 95% CI, 2.3 to 9.2). In the sensitivity analyses restricted to women with APP measurement data, the association became slightly stronger (Table S3).

**Table 2:** Odds ratio of stillbirth

Characteristics	All (56 cases)				Unexplained (48 cases)			
	Raw odds ratio (95% CI)	*P* value	Adjusted odds ratio (95% CI)	*P* value	Raw odds ratio (95% CI)	*P* value	Adjusted odds ratio (95% CI)	*P* value
Placental perfusion								
Normal	1.0		1.0		1.0		1.0	
Insufficiency	4.8 (2.5–9.1)	< 0.001	4.2 (2.2–8.0)	< 0.001	5.3 (2.7–10.5)	< 0.001	4.6 (2.3–9.2)	< 0.001
NA	1.7 (0.9–3.2)	0.10	1.6 (0.9–3.1)	0.13	1.6 (0.8–3.3)	0.16	1.6 (0.8–3.1)	0.21
Maternal age at delivery (years)								
< 25	1.0		1.0		1.0		1.0	
25–34	0.9 (0.3–3.0)	0.91	0.9 (0.3–2.9)	0.86	0.8 (0.2–2.5)	0.67	0.7 (0.2–2.4)	0.60
≥ 35	1.0 (0.3–4.1)	0.98	0.8 (0.2–3.4)	0.77	1.0 (0.3–4.1)	0.98	0.8 (0.2–3.3)	0.71
Residence								
Local	1.0		1.0		1.0		1.0	
Nonlocal	1.1 (0.6–2.0)	0.74	1.1 (0.6–2.0)	0.81	1.1 (0.6–2.1)	0.75	1.1 (0.5–2.1)	0.85
Parity								
Nulliparous	1.0		1.0		1.0		1.0	
Pluriparous	1.2 (0.6–2.4)	0.57	1.3 (0.6–2.7)	0.51	1.3 (0.6–2.7)	0.49	1.3 (0.6–2.9)	0.48
Assisted conception								
No	1.0		1.0		1.0		1.0	
Yes	1.0 (0.1–7.2)	0.99	0.9 (0.1–6.9)	0.94	1.2 (0.2–8.4)	0.88	1.0 (0.1–7.8)	0.98
Gestational diabetes mellitus								
No	1.0		1.0		1.0		1.0	
Yes	1.1 (0.4–2.7)	0.89	0.9 (0.4–2.4)	0.87	1.3 (0.5–3.2)	0.62	1.1 (0.4–2.8)	0.88
Preeclampsia								
No	1.0		1.0		1.0		1.0	
Yes	4.5 (2.4–8.6)	< 0.001	3.8 (2.0–7.4)	< 0.001	4.9 (2.5–97)	< 0.001	4.0 (2.0–8.1)	< 0.001
Sex								
Male	1.0		1.0		1.0		1.0	
Female	1.9 (1.1–3.3)	0.020	1.9 (1.1–3.3)	0.020	1.9 (1.1–3.5)	0.028	2.0 (1.1–3.5)	0.027

**Fig. 1:**
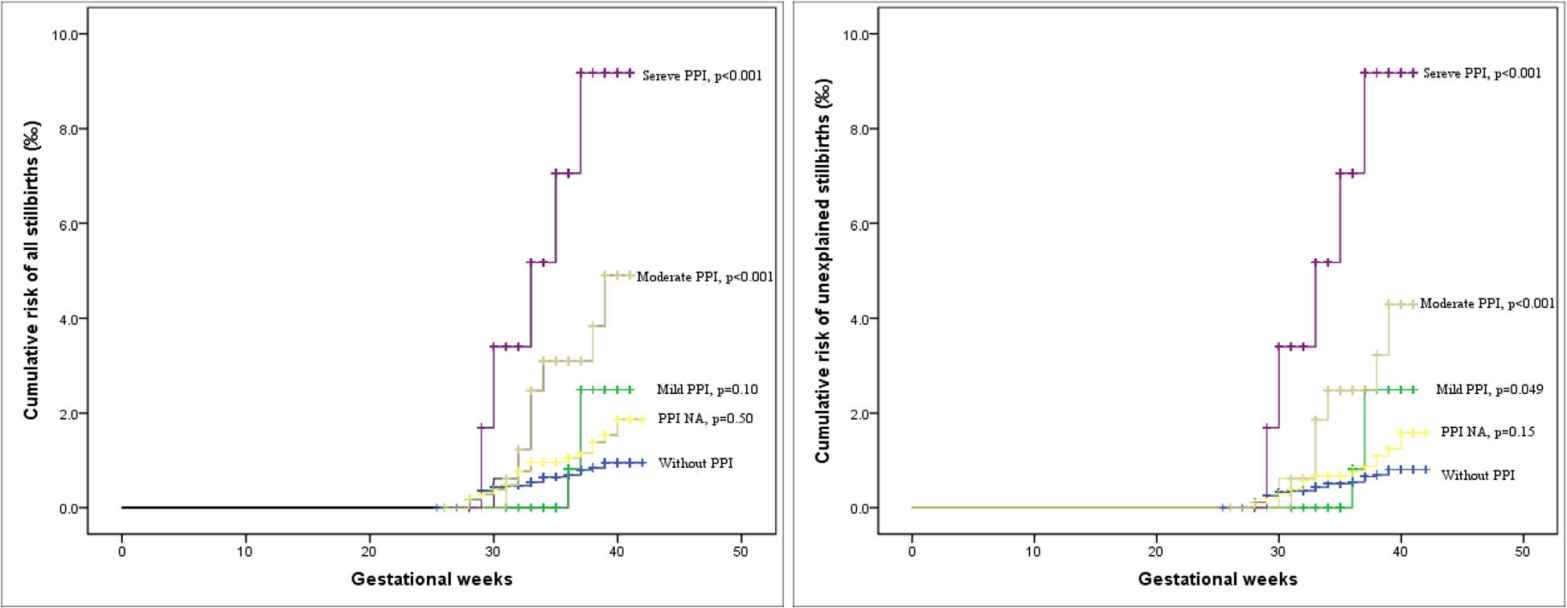
Cumulative risk of stillbirth (all and unexplained) across the groups by the number of placental perfusion insufficiency markers^*^. ^*^ APP NA, placental perfusion insufficiency data not available, *P* values for the log-rank (Mantel-Cox) method compared with the cumulative risk of stillbirth in the group without APP exposure

The association strengths were consistent across groups of infants exposed to variants of APP that was defined by abnormality in left or right UtA-PI or UtA-RI, and the ORs ranged from 3.2 to 5.3. However, no significant higher risk of stillbirths was found for infants exposed to a separate presence of right or left EDN (Table 3). The risk of stillbirth increased with the severity of APP (trend *P* < 0.001). The cumulative risks of all and unexplained stillbirths in the moderate and severe APP groups were significantly higher than those in the perfusion normal group (both *P* < 0.001) (Table 3). The foetuses exposed to mild APP also showed a stronger and statistically significantly higher risk of unexplained stillbirth than the foetuses of the mothers with normal perfusion (*P* = 0.049) (Fig. 1).

**Table 3:** Variant in placental perfusion insufficiency and odds ratio of stillbirth

Variant of placental perfusion insufficiency		All (56 cases)					Unexplained (48 cases)				
		No. of infants	No. of cases	Prevalence (‰) (95% CI)	Adjusted odds ratio (95% CI) ^a^	*P* value	No. of infants	No. of cases	Prevalence (‰) (95% CI)	Adjusted odds ratio (95% CI) ^a^	*P* value
Right uterine artery											
PI	Normal (≤P95)	29,709	31	1.0 (0.7–1.4)	1.0		29,704	26	0.9 (0.5–1.2)	1.0	
	High (>P95)	1560	9	5.8 (2.0–9.5)	3.9 (1.8–8.6)	0.001	1560	9	5.8 (2.0–9.5)	4.8 (2.1–10.7)	< 0.001
	NA	10,371	16	1.5 (0.8–2.3)	1.4 (0.8–2.6)	0.27	10,368	13	1.3 (0.6–1.9)	1.3 (0.7–2.6)	0.39
RI	Normal (≤P95)	29,881	33	1.1 (0.7–1.5)	1.0		29,876	28	0.9 (0.6–1.3)	1.0	
	High (>P95)	1388	7	5.0 (1.3–8.8)	3.2 (1.4–7.5)	0.008	1388	7	5.0 (1.3–8.8)	3.8 (1.6–9.1)	0.003
	NA	10,371	16	1.5 (0.8–2.3)	1.3 (0.7–2.4)	0.35	10,368	13	1.3 (0.6–1.9)	1.3 (0.6–2.5)	0.50
EDN	No	31,011	39	1.3 (0.9–1.7)	1.0		31,006	34	1.1 (0.7–1.5)	1.0	
	Yes	275	1	3.6 (0.0–10.8)	1.5 (0.2–11.5)	0.70	275	1	3.6 (0.0–10.8)	1.8 (0.2–13.7)	0.58
	NA	10,354	16	1.5 (0.8–2.3)	1.2 (0.7–2.1)	0.58	10,351	13	1.3 (0.6–1.9)	1.1 (0.6–2.1)	0.81
Left uterine artery											
PI	Normal (≤P95)	29,660	31	1.0 (0.7–1.4)	1.0		29,656	27	0.9 (0.6–1.3)	1.0	
	High (>P95)	1566	9	5.7 (2.0–9.5)	4.0 (1.8–8.7)	0.001	1565	8	5.1 (1.6–8.6)	4.1 (1.8–9.4)	0.001
	NA	10,414	16	1.5 (0.8–2.3)	1.4 (0.8–2.6)	0.28	10,411	13	1.2 (0.6–1.9)	1.3 (0.7–2.5)	0.47
RI	Normal (≤P95)	19,759	30	1.0 (0.6–1.4)	1.0		29,755	26	0.9 (0.5–1.2)	1.0	
	High (>P95)	1467	10	6.8 (2.6–11.0)	5.1 (2.4–10.8)	< 0.001	1466	9	6.1 (2.1–10.1)	5.3 (2.4–11.8)	< 0.001
	NA	10,414	16	1.5 (0.8–2.3)	1.5 (0.8–2.7)	0.23	10,411	13	1.2 (0.6–1.9)	1.3 (0.7–2.6)	0.39
EDN	No	30,891	40	1.3 (0.9–1.7)	1.0		30,886	35	1.1 (0.8–1.5)	1.0	
	Yes	393	0	–	–		393	0	–	–	
	NA	10,356	16	1.5 (0.8–2.3)	1.1 (0.6–2.1)	0.66	10,353	13	1.3 (0.6–1.9)	1.0 (0.5–2.0)	0.9
No. of insufficiency markers											
0		27,724	25	0.9 (0.5–1.3)	1.0		27,720	21	0.8 (0.4–1.1)	1.0	
1		1263	3	2.4 (0.0–5.1)	2.4 (0.7–8.1)	0.15	1263	3	2.4 (0.0–5.1)	2.9 (0.9–9.9)	0.083
2		1626	7	4.3 (1.1–7.5)	4.1 (1.8–9.7)	0.001	1625	6	3.7 (0.7–6.6)	4.2 (1.7–10.6)	0.002
3 and above		596	5	8.4 (1.0–15.7)	5.3 (1.9–15.2)	0.002	596	5	8.4 (1.0–15.7)	6.5 (2.2–19.0)	0.001
NA		10,431	16	1.5 (0.8–2.3)	1.6 (0.9–3.0)	0.14	10,428	13	1.2 (0.6–1.9)	1.5 (0.8–3.1)	0.23

Abbreviations: *PI* Pulsatility index, *RI* Resistance index, *EDN* Early diastolic notching

^a^ Adjusted for maternal age at delivery (< 25, 25–34, or ≥ 35), residence (local or nonlocal), parity (nulliparous or pluriparous), gestational diabetes mellitus (yes or no), assisted conception (yes or no), preeclampsia (yes or no) and sex (male or female)

### Effect of APP on the risk of stillbirth

APP itself directly mediated the risk of stillbirth and acted as an intermediate factor in the aetiological chain between PE and stillbirth. In total, 59.0% of the risk of stillbirth could be attributed to APP. A similar effect of APP on the risk of stillbirth was found for female foetuses, accounting for 58.0% of the risk. However, APP was the only factor responsible for 94.5% of the risk of stillbirth for male foetuses, while PE did not contribute significantly (Table 4).

**Table 4:** Estimated size of the effect of placental perfusion insufficiency on the risk of unexplained stillbirth stratified by sex (%)^a^

Sex	Direct factor ^b^	Total effect			The effect of PE			The effect of placental perfusion insufficiency			
		Coef.	Standard Error	*P* value	Coef.	Standard Error	*P* value	Coef.	Standard Error	*P* value	Size of the total effect (%)
Male	APP	1.45	0.54	0.008	0.08	0.09	0.42	1.37	0.56	0.015	94.5
	PE	1.11	0.60	0.062	0.87	0.61	0.15	0.24	0.11	0.038	21.6
	Combined	2.56						1.61			62.9
Female	APP	1.83	0.54	0.001	0.15	0.10	0.12	1.68	0.59	0.004	91.8
	PE	1.60	0.52	0.002	1.28	0.55	0.02	0.31	0.12	0.008	19.4
	Combined	3.43						1.99			58.0
Total	APP	1.68	0.36	< 0.001	0.12	0.07	0.10	1.56	0.37	< 0.001	92.9
	PE	1.44	0.43	0.001	1.16	0.44	0.009	0.28	0.07	< 0.001	19.4
	Combined	3.12						1.84			59.0

^a^ Adjusted for maternal age at delivery (< 25, 25–34, or ≥ 35), residence (local or nonlocal), parity (nulliparous or pluriparous), gestational diabetes mellitus (yes or no), assisted conception (yes or no), preeclampsia (yes or no) and sex (male or female) in total. Sex was not included in the models of the stratification analyses

^b^ Direct factor refers to the factor associated with the risk of disease; indirect factor refers to the factor that may be involved in the aetiological chain of the direct factor and the occurrence of disease and that plays a role in the risk of disease

### Performance in screening for stillbirths

The ROC curve analyses indicated that the number of weeks between the placental perfusion measurement and delivery had a significant value in predicting stillbirth at the criterion value range of 3 to 14.6 weeks. The stratification analyses showed that in a similar criterion range, the number of weeks between the two dates performed best in screening for stillbirth among women with normal placental perfusion and male foetuses (Table 5). Women with normal placental perfusion and a male foetus exhibited the highest specificity (approximately 94%, range from 93.37 to 94.05%) at any given false negative rate between 1 and 10% (Table 6).

**Table 5:** Performance of the number of weeks between the placental perfusion measurement and delivery in screening for unexplained stillbirths under regular and assumed conditions

Stratification	Area under the curve (95% CI)	Initiation time (weeks)	Regular condition			Assumed condition ^a^		
			Criterion value (weeks)	Sensitivity (% (95% CI))	Specificity (% (95% CI))	Criterion value (weeks)	Sensitivity (% (95% CI))	Specificity (% (95% CI))
Placental perfusion								
Normal group	0.948 (0.945–0.951)	≥ 3	≤ 15.0	90.5 (69.6 ~ 98.8)	83.4 (83.0–83.9)	≤ 11.8	66.7 (43.0 ~ 85.4)	98.8 (98.7–99.0)
APP group	0.867 (0.855–0.878)	≥ 5	≤ 14.6	85.7 (57.2 ~ 98.2)	84.4 (83.1–85.5)	≤ 14.0	78.6 (49.2 ~ 95.3)	89.4 (88.3–90.4)
Sex								
Male	0.972 (0.969–0.974)	≥ 3	≤ 14.6	100 (76.8–100)	89.03 (88.5–89.5)	≤ 8.3	50 (23.0–77.0)	99.7 (99.6–99.7)
Female	0.895 (0.890–0.900)	≥ 4	≤ 15.0	80.9 (58.1–94.6)	83.66 (83.1–84.2)	≤ 11.9	61.9 (38.4–81.9)	98.7 (98.5–98.9)
Combination								
Normal and male	0.985 (0.983–0.987)	≥ 3	≤ 14.1	100 (66.4–100)	93.3(92.9–93.7)	≤ 8.3	66.7 (29.9–92.5)	99.7 (99.6–99.8)
Normal and female	0.924 (0.920–0.929) ^†^	≥ 4	≤ 15.4	91.7 (61.5–99.8)	76.41 (75.7–77.1)	≤ 11.3	58.3 (27.7–84.8)	99.2 (99.0–99.3)
APP and male	0.920 (0.906–0.931) ^†^	≥ 5.1	≤ 14.6	100 (47.8–100)	84.0 (82.2–85.6)	≤ 14.6	100 (47.8–100)	84.0 (82.2–85.6)
APP and female	0.839 (0.820–0.857)	≥ 5	≤ 14.0	77.8 (40.0–97.2)	89.5 (87.9–90.9)	≤ 11.9	66.7 (29.9–92.5)	96.8 (95.8–97.6)

^a^ Assumed condition: the cost of a false negative decision was 100 times that of a false positive decision (cost of false positive decision was 1, cost of false negative decision was 100, and the costs of both a true positive and negative decision were 0), and the rate of unexplained stillbirths in the sample reflects the real prevalence of stillbirth in the population

^†^ *P* < 0.05 compared with the normal and male groups

**Table 6:** Criterion range and specificity of weeks after placental perfusion examination in screening for unexplained stillbirths at given false negative rates

Stratification	False negative rate							
	1.0%		2.5%		5.0%		10.0%	
	Criterion range (weeks)	Specificity (%, (95% CI))	Criterion range (weeks)	Specificity (%, (95% CI))	Criterion range (weeks)	Specificity (%, (95% CI))	Criterion range (weeks)	Specificity (%, (95% CI))
Placental perfusion								
Normal group	3.0–15.7	66.89 (65.24–83.29)	3.0–15.7	68.26 (65.61–83.74)	3.0–15.4	75.08 (67.13–90.24)	3.0–15.0	83.58 (67.65–93.62)
APP group	5.0–17.6	11.29 (9.65–61.00)	5.0–17.6	11.85 (9.89–62.43)	5.0–17.5	12.78 (10.31–84.98)	5.0–17.1	61.39 (11.19–89.62)
Sex								
Male	3.0–14.6	89.24 (88.47–92.89)	3.0–14.6	89.55 (88.57–93.27)	3.0–14.6	90.06 (88.75–93.73)	3.0–14.1	93.02 (89.17–94.63)
Female	4.0–17.6	13.87 (12.94–67.78)	4.0–17.5	14.66 (13.21–68.94)	4.0–15.7	67.17 (13.95–83.56)	4.0–15.7	69.29 (14.55–84.20)
Combination								
Normal and male	3.0–14.1	93.37 (92.65–95.09)	3.0–14.1	93.49 (92.76–95.22)	3.0–14.1	93.68 (92.90–95.47)	3.0–14.0	94.05 (93.03–95.90)
Normal and female	4.0–15.7	68.28 (66.92–77.05)	4.0–15.7	69.02 (67.17–84.48)	4.0–15.7	70.27 (67.49–84.85)	4.0–15.4	77.06 (68.01–85.60)
APP and male	5.1–14.6	84.06 (81.53–89.19)	5.1–14.6	84.17 (81.64–89.37)	5.1–14.6	84.35 (81.83–89.49)	5.1–14.5	84.72 (82.21–89.91)
APP and female	5.0–17.6	12.32 (9.89–61.80)	5.0–17.6	12.60 (10.03–62.36)	5.0–17.5	13.06 (10.16–63.35)	5.0–17.5	13.98 (10.41–66.22)

## Discussion

The present study identified that stillbirth risk was associated with APP (e.g., right PI > 1.33 or right RI > 0.69 or left PI > 1.39 or left RI > 0.70 at gestational week 22) and increased with the severity of APP. The risk of stillbirth is predominantly attributed to APP, in contrast to the effect of PE. Furthermore, different paths towards stillbirth may exist between sexes: an APP-mediated pathway without PE involvement was found for male foetuses, while an additional PE-mediated and APP-modified pathway was also revealed for female foetuses.

We extended the findings of previous studies [1, 2, 4, 21] by quantifying the dominant role of APP in the risk of stillbirth. In the quantitative model, we found that 59% of the risk of stillbirth may be attributed to APP. The proportion was within the range of 23 to 65% observed in previous studies [2, 22, 23]. The hypothesis that multiple mechanisms may lead to stillbirth was validated since at least two mechanisms were found, including one by APP and the other by PE and/or its potential mechanism.

Impaired uteroplacental circulation is closely related to placental dysfunction and plays a central role in the pathogenesis of neonatal complications, such as preterm delivery, PE and foetal growth restriction [7, 8, 24, 25], which may increase the risk of stillbirth [1, 2]. Moreover, APP may impair oxygen transport and reduce the oxygen supply to the foetal vasculature [26–28], which is associated with increased placental apoptosis and accelerated ageing of the placenta [25, 29, 30], resulting in compromised foetal viability [26, 30].

Although the relationship between sex and stillbirth disappeared in the sensitivity analysis, probably due to the insufficient sample size, we found that female foetuses had an increased risk of stillbirth compared to males, which is consistent with previous studies of the Chinese population [31, 32]. However, a reversed trend was found in a meta-analysis in which the authors argued that the heterogeneity may be plausibly explained by the intervention against female foetuses as a means of prenatal sex selection [33]. Sex bias might actually exist in some areas of China, but in our study, we can exclude the impact of such bias on the association between sex and stillbirth because no stillbirth cases were due to sex selection. Foetal sex affects early placentation processes and placental function and may play a leading role in the development of subsequent complications through different mechanisms, such as PE [34–36]. These mechanisms associated with PE might cause sex differences in stillbirth since we found that PE was involved in the development of stillbirth for female foetuses but not for male foetuses. Male foetuses may be exposed to higher levels of intrauterine hypertension and APP for a longer period of time [36, 37], and they might compensate to adapt to APP and improve survival at the cost of certain organ damage, such as congenital urogenital anomalies [9, 14]. Ethnicity disparity may partly contribute to the heterogeneity of the sex-stillbirth relationship between Asians and non-Asians, similar to the association between sex and PE [37].

Previous studies have shown that screening models (UtA-PI alone or combined with maternal factors, foetal biometry, and PlGF) had higher detection rates of stillbirth in foetuses aged < 32 weeks of gestation compared with stillbirth in foetuses aged ≥37 weeks of gestation [9, 10]. Similarly, we found that the criterion value for male foetuses differed from that for female foetuses (8.3 vs. 11.9 weeks after the placental perfusion measurement, respectively; or 30.9 vs. 34.6 weeks of gestation, respectively) under the assumed conditions. The gestational week differences and sex disparity in screening for stillbirth may be associated with PE since 34 weeks of gestation was the criterion value for classifying early- and late-onset PE, and PE was involved in the development of stillbirth in female foetuses but not male foetuses, as revealed in the present study.

Our findings refute the myth that stillbirths are inevitable and establish a more effective method for the screening of stillbirths. The relationship between APP and the risk of stillbirth illustrates the necessity and importance of placental perfusion measurement. Therefore, publicizing the importance of placental perfusion measurements in the second trimester should be improved in the field of obstetrics and among pregnant women so that coverage of this measurement could be increased. More attention should be paid to those at high risk of stillbirth, such as women with moderate APP or with any singular abnormality in the left or right UtA-RI or UtA-PI (details of parameters for UtA by gestational week are displayed in Table S2). Second, stratification by sex and placental perfusion may effectively improve performance in screening for stillbirth. Clinicians and women of this subgroup could not worry about the risk of stillbirth during the period. In contrast, measures, such as shortening the prenatal examination interval and continuous dynamic monitoring of foetuses, should be adopted after placental perfusion measurement to detect an abnormal foetus in a timely manner and balance the risk by early induction or caesarean section, especially for women with APP and a female foetus. Finally, improving prevention awareness of stillbirth and compliance with antenatal care is important since a woman’s empowerment plays an important role in reducing stillbirths [38], and most unexplained stillbirths (74.4%, 35/47) in the present study were found and reported by the mothers.

Our study has certain limitations. First, the stillbirth rate was low, and it was impossible to determine whether this was due to high-quality obstetric examinations and care at the hospital, the exclusion of specific mothers who might have had a higher risk of stillbirth (e.g., those lost to follow-up) or both. However, the stillbirth rate may be representative at the regional maternal health care level [39], so this potential underestimation will not affect the results. Second, although the sample size was relatively large, the sample size seemed to not have enough power in the sensitivity analysis, in which the 25.1% of women who did not take part in the placental perfusion measurement were excluded and in which the association between sex and the risk of stillbirth found in the multivariable analysis disappeared. Studies with larger sample sizes that examine the sex disparity of stillbirth are warranted. Finally, this was a single-centre study of pregnant women in Shanghai, and we mainly included causes of stillbirth, namely, maternal age, pregnancy complications (e.g., GDM and PE), assisted conception, foetal sex, congenital malformations (accounting for 2.1% [1/48] to 16.1% [assuming all 9 congenital anomalies led to stillbirth before induction of labour]) and placental condition (APP); however, we did not include maternal infections (e.g., malaria and syphilis), which are common causes of stillbirth in low-income countries [1, 4, 21]. Moreover, the rate of unexplained stillbirths was high (85.7%, 48/56), and the causes of the unexplained cases could not be further classified since a multidisciplinary review and placental pathology examination were not performed. Therefore, the generalizability of these findings may be limited in low- and middle-income countries.

## Conclusion

In conclusion, stillbirth risk was associated with APP and increased with the severity of APP measured in the second trimester. APP is a major cause of stillbirth, and related measures should be taken to reduce this risk. Different mechanisms of foetal stillbirth were found between male and female foetuses, and further studies are warranted to elucidate the reasons for sex disparities.

## Supplementary Information

**Additional file 1: Table S1**. Details of 56 stillbirths. **Table S2**. The median and ninety-fifth percentile (P95) of the values of the left and right uterine artery pulsatility index (PI) and resistance index (RI) by gestational week. **Table S3**. Odds ratio of stillbirths in pregnant women with placental perfusion measurement data.

## Data Availability

The datasets used in the present study are available from the corresponding author (wjnhmm@126.com) on reasonable request only.

## Abbreviations

APP: Abnormal placental perfusion
CI: Confidence interval
GDM: Gestational diabetes mellitus
OR: Odds ratio
PE: Preeclampsia
PI: Pulsatility index
RI: Resistance index
UtA: Uterine artery
EDN: Early diastolic notching
