# Supplementary material for: Abnormal placental perfusion and the risk of stillbirth: a hospital-based retrospective cohort study
Source: BMC Pregnancy Childbirth. 2021 Apr 17;21:308. doi: 10.1186/s12884-021-03776-8 (PMC8052678; doi:10.1186/s12884-021-03776-8)
Supplement: Supplementary file 1 — Additional file 1: Table S1. Details of 56 stillbirths. Table S2. The median and ninety-fifth percentile (P95) of the values of the left and right uterine artery pulsatility index (PI) and resistance index (RI) by gestational week. Table S3. Odds ratio of stillbirths in pregnant women with placental perfusion measurement data. [file 12884_2021_3776_MOESM1_ESM.docx]

Table S1. Details of 56 stillbirths^#^

| No. | PE (1 yes; 0 no) | Gestational weeks at Doppler scan | Placental perfusion insufficiency | | | Unexplained Stillbirth (1 yes, 0 no) | Reasons for hospitalization ^*^ | Gestational weeks at delivery | Natural labor (1yes, 0no) | Gender (1male, 0female) | Birth weight (g) |
| --- | --- | --- | --- | --- | --- | --- | --- | --- | --- | --- | --- |
|  |  |  | 1yes;  0 no | No. of abnormal markers | Specific abnormal marker |  |  |  |  |  |  |
| 1 | 1 | 22+5 | 0 | 0 |  | 1 | 1 | 34 | 1 | 0 | 2290 |
| 2 | 0 | NA | NA | NA |  | 1 | 4 | 31 | 1 | 0 | 830 |
| 3 | 0 | 22 | 0 | 0 |  | 1 | 1 | 30 | 1 | 1 | 1740 |
| 4 | 0 | 23 | 1 | 4 | Right PI/RI, left PI/RI | 1 | 1 | 37 | 1 | 1 | 3700 |
| 5 | 0 | NA | NA | NA |  | 1 | 1 | 38 | 1 | 0 | 3060 |
| 6 | 0 | 22+3 | 0 | 0 |  | 1 | 1 | 28 | 1 | 1 | 725 |
| 7 | 0 | 22+1 | 1 | 1 | Right PI | 1 | 3+2 | 36 | 1 | 1 | 2280 |
| 8 | 0 | NA | NA | NA |  | 1 | 2 | 28 | 1 | 0 | 1040 |
| 9 | 0 | 22+1 | 1 | 3 | Right EDN, right PI/RI | 1 | 1 | 33 | 1 | 0 | 1910 |
| 10 | 1 | NA | NA | NA |  | 1^†^ | 2 | 38 | 1 | 0 | 2520 |
| 11 | 0 | 22 | 0 | 0 |  | 0 | 5 | 28 | 1 | 1 | 1310 |
| 12 | 1 | 22+5 | 0 | 0 |  | 0/1^‡^ | 5 | 31 | 1 | 1 | 1150 |
| 13 | 0 | 21+4 | 1 | 2 | Left PI/RI | 1 | 1+3 | 33 | 0 | 0 | 1770 |
| 14 | 1 | NA | NA | NA |  | 1 | 1 | 32 | 1 | 0 | 730 |
| 15 | 0 | 22+1 | 1 | 2 | Left PI/RI | 1 | 1 | 34 | 1 | 0 | 2390 |
| 16 | 0 | NA | NA | NA |  | 0 | 5 | 32 | 1 | 0 | 390 |
| 17 | 0 | 23 | 1 | 1 | Right PI | 1 | 1 | 37 | 1 | 0 | 2530 |
| 18 | 1 | NA | NA | NA |  | 0 | 5 | 28 | 1 | 0 | 690 |
| 19 | 0 | 23+1 | 0 | 0 |  | 1 | 1 | 33 | 1 | 0 | 2010 |
| 20 | 1 | 23 | 1 | 2 | Right PI/RI | 1 | 3 | 33 | 0 | 0 | 1267 |
| 21 | 0 | 22+3 | 0 | 0 |  | 1 | 3+4 | 37 | 1 | 0 | 2950 |
| 22 | 0 | NA | NA | NA |  | 1 | 1 | 33 | 1 | 1 | 2280 |
| 23 | 0 | 21+3 | 1 | 2 | Right PI/RI | 1 | 1 | 39 | 1 | 0 | 2760 |
| 24 | 1 | 22+2 | 1 | 4 | Right PI/RI, left PI/RI | 1 | 3 | 35 | 1 | 1 | 2180 |
| 25 | 0 | 23+2 | 0 | 0 |  | 0 | 5 | 28 | 1 | 0 | 1110 |
| 26 | 0 | 22 | 0 | 0 |  | 1 | 3 | 37 | 1 | 0 | 2570 |
| 27 | 0 | 22+3 | 0 | 0 |  | 1 | 1 | 30 | 1 | 1 | 1220 |
| 28 | 0 | 22+3 | 1 | 1 | Left RI | 1 | 4 | 37 | 1 | 1 | 1900 |
| 29 | 0 | 21+1 | 0 | 0 |  | 1 | 2 | 29 | 1 | 1 | 1230 |
| 30 | 0 | 21+3 | 0 | 0 |  | 0 | 5 | 29 | 0 | 0 | 1020 |
| 31 | 0 | 22+3 | 0 | 0 |  | 1 | 1 | 33 | 1 | 0 | 2300 |
| 32 | 0 | 22+6 | 0 | 0 |  | 1 | 3 | 37 | 1 | 1 | 4100 |
| 33 | 0 | 21+3 | 0 | 0 |  | 1 | 1 | 28 | 1 | 0 | 1250 |
| 34 | 0 | NA | NA | NA |  | 1 | 1 | 36 | 1 | 0 | 2110 |
| 35 | 0 | 23+2 | 0 | 0 |  | 1 | 1 | 39 | 1 | 0 | 2680 |
| 36 | 0 | NA | NA | NA |  | 1 | 1 | 39 | 1 | 0 | 2860 |
| 37 | 0 | NA | NA | NA |  | 1 | 2 | 37 | 0 | 1 | 3870 |
| 38 | 0 | 22+4 | 0 | 0 |  | 1 | 2 | 29 | 1 | 0 | 1550 |
| 39 | 0 | 22+2 | 1 | 2 | Left PI/RI | 1 | 2 | 30 | 1 | 0 | 1230 |
| 40 | 0 | 22+5 | 1 | 4 | Right PI/RI, left PI/RI | 1 | 2 | 30 | 1 | 1 | 950 |
| 41 | 1 | 23+2 | 1 | 4 | Right PI/RI, left PI/RI | 1 | 2 | 29 | 1 | 0 | 610 |
| 42 | 0 | 22+5 | 0 | 0 |  | 0 | 5 | 34 | 1 | 0 | 2900 |
| 43 | 1 | NA | NA | NA |  | 1 | 1 | 29 | 1 | 0 | 960 |
| 44 | 1 | 23 | 0 | 0 |  | 1 | 2 | 38 | 1 | 0 | 3600 |
| 45 | 0 | 22+2 | 0 | 0 |  | 1 | 3 | 34 | 1 | 1 | 2690 |
| 46 | 0 | NA | NA | NA |  | 0 | 5 | 33 | 1 | 1 | 2400 |
| 47 | 0 | 22+2 | 1 | 2 | Left PI/RI | 1 | 1 | 38 | 1 | 0 | 2800 |
| 48 | 0 | NA | NA | NA |  | 1 | 1 | 32 | 1 | 0 | 1915 |
| 49 | 0 | 22+1 | 0 | 0 |  | 1 | 1 | 36 | 0 | 1 | 2660 |
| 50 | 0 | 23+4 | 0 | 0 |  | 1 | 1 | 39 | 1 | 0 | 2840 |
| 51 | 1 | NA | NA | NA |  | 1 | 2 | 30 | 1 | 1 | 960 |
| 52 | 0 | 23+1 | 1 | 2 | Left PI/RI | 0 | 5 | 32 | 1 | 1 | 1620 |
| 53 | 1 | 22+1 | 0 | 0 |  | 1 | 2 | 29 | 1 | 0 | 990 |
| 54 | 0 | 22+6 | 0 | 0 |  | 1 | 3 | 28 | 1 | 0 | 980 |
| 55 | 0 | NA | NA | NA |  | 1 | 1 | 40 | 1 | 0 | 3810 |
| 56 | 0 | 22+3 | 0 | 0 |  | 1 | 2 | 29 | 1 | 1 | 1180 |

^*^ 1, self-reported abnormal fetal status (e.g., decreased or disappeared fetal motility); 2,abnormal fetal status detected by prenatal examination; 3, unknown causes of abdominal pain; 4,vaginal fluids or bleeding; 5, termination of pregnancy due to the discovery of severe congenital anomalies.

^†^ intrapartum stillbirth case; the remaining unexplained cases were antepartum stillbirth.

^‡^ Unexplained stillbirth occurred before the induction of labor during the termination of pregnancy due to disorders related to fetal growth.

^#^ Maternal age at delivery ranged from 24 to 45 years.

Table S2. The median and ninety-fifth percentile (P95) of the values of the left and right uterine artery pulsatility index (PI) and resistance index (RI) by gestational week

| Weeks at measurement | No. of women | Right PI | | Right RI | | Left PI | | Left RI | |
| --- | --- | --- | --- | --- | --- | --- | --- | --- | --- |
|  |  | Median | P95 | Median | P95 | Median | P95 | Median | P95 |
| 20 | 362 | 0.83 | 1.52 | 0.53 | 0.73 | 0.83 | 1.47 | 0.52 | 0.71 |
| 21 | 2807 | 0.82 | 1.44 | 0.52 | 0.71 | 0.85 | 1.51 | 0.53 | 0.73 |
| 22 | 18,064 | 0.79 | 1.33 | 0.51 | 0.69 | 0.82 | 1.39 | 0.52 | 0.70 |
| 23 | 9426 | 0.78 | 1.31 | 0.51 | 0.69 | 0.80 | 1.37 | 0.52 | 0.69 |
| 24 | 556 | 0.76 | 1.35 | 0.51 | 0.69 | 0.81 | 1.34 | 0.52 | 0.69 |

Table S3. Odds ratio of stillbirths in pregnant women with placental perfusion measurement data^*^

| Characteristics | | All (40 cases) | | Unexplained (35 cases) | |
| --- | --- | --- | --- | --- | --- |
|  |  | Adjusted odds ratio (95% CI) | P value | Adjusted odds ratio (95% CI) | P value |
| Placental perfusion | |  |  |  |  |
|  | Normal | 1.0 |  | 1.0 |  |
|  | Insufficiency | 4.4 (2.3-8.4) | <0.001 | 4.7 (2.4-9.5) | <0.001 |
| Maternal age at delivery (years) | |  |  |  |  |
|  | <25 | 1.0 |  | 1.0 |  |
|  | 25-34 | 0.9 (0.2-3.7) | 0.85 | 0.7 (0.2-3.1) | 0.67 |
|  | ≥ 35 | 0.7 (0.1-4.6) | 0.73 | 0.6 (0.1-4.2) | 0.65 |
| Residence | |  |  |  |  |
|  | Local | 1.0 |  | 1.0 |  |
|  | Nonlocal | 1.1 (0.5-2.4) | 0.75 | 1.1 (0.5-2.5) | 0.77 |
| Parity | |  |  |  |  |
|  | Nulliparous | 1.0 |  | 1.0 |  |
|  | Pluriparous | 1.0 (0.4-2.6) | 0.96 | 1.1 (0.4-3.1) | 0.82 |
| Gestational diabetes mellitus | |  |  |  |  |
|  | No | 1.0 |  | 1.0 |  |
|  | Yes | 0.9 (0.3-2.9) | 0.82 | 1.0 (0.3-3.3) | 0.98 |
| Preeclampsia | |  |  |  |  |
|  | No | 1.0 |  | 1.0 |  |
|  | Yes | 2.8 (1.2-6.4) | 0.016 | 3.2 (1.4-7.5) | 0.007 |
| Sex | |  |  |  |  |
|  | Male | 1.0 |  | 1.0 |  |
|  | Female | 1.6 (0.9-3.0) | 0.140 | 1.6 (0.8-3.2) | 0.17 |

^*^ Assisted conception (yes or no) was not included in the regression model since there was no stillbirth case in an assisted conception pregnancy.
